# A Simple One-Step Modification of Shrimp Shell for the Efficient Adsorption and Desorption of Copper Ions

**DOI:** 10.3390/molecules26185690

**Published:** 2021-09-20

**Authors:** Changkun Liu, Hui Wen, Kaili Chen, Yanni Chen

**Affiliations:** College of Chemistry and Environmental Engineering, Shenzhen University, Shenzhen 518060, China; 2070223047@email.szu.edu.cn (H.W.); 2010140078@email.szu.edu.cn (K.C.); 2010140077@email.szu.edu.cn (Y.C.)

**Keywords:** waste shrimp shells, adsorption experiment, desorption experiment, modifier recycling

## Abstract

Removing toxic heavy metal species from aqueous solutions is a point of concern in our society. In this paper, a promising biomass adsorbent, the modified waste shrimp shell (MS), for Cu (II) removal was successfully prepared using a facile and simple one-step modification, making it possible to achieve high-efficiency recycling of the waste NaOH solution as the modification agent. The outcome shows that with the continuous increase in pH, temperature and ion concentration, the adsorption effect of MS on Cu (II) can also be continuously improved. Adsorption isotherm and adsorption kinetics were fitted with the Langmuir isotherm model and the pseudo-second-order model, respectively, and the maximum adsorption capacity of Cu (II) as obtained from the Langmuir isotherm model fitting reached 1.04 mmol/g. The systematic desorption results indicated that the desorption rate of Cu (II) in the MS could reach 100% within 6 min, where HNO_3_ is used as the desorption agent. Moreover, experiments have proven that after five successive recycles of NaOH as a modifier, the adsorption capacity of MS on Cu (II) was efficient and stable, maintaining tendency in 0.83–0.85 mmol/g, which shows that waste NaOH solution can be used as a modification agent in the preparation of waste shrimp shell adsorbent, such as waste NaOH solution produced in industrial production, thereby making it possible to turn waste into renewable resources and providing a new way to recycle resources.

## 1. Introduction

The large-scale use of copper as a heavy metal material in production activities such as copper mining, smelting and processing is a key factor that has led to increasingly serious pollution of Cu (II) in water circulation. Due to its bioaccumulation and toxicity even at low concentrations, large amounts of Cu (II) can accumulate in the human body through the food chain and react with proteins and enzymes to bring about acute poisoning, subsequently leading to various diseases and functional disorders, which can cause serious harm to people’s health [1,2,3,4,5]. Therefore, the efficient removal of Cu (II) is a hot point in the current research. Currently, the main methods used in removing heavy metal pollution include adsorption [1,6], ion exchange [7], precipitation [8] and membrane process [9]. However, these technologies have not been applied on a large scale owing to the economic cost limitations and complex processing technology. Comparatively speaking, adsorption has attracted researchers’ attention due to its versatility in design and application, ease of handing and relatively low cost [10,11]. Particularly, studies on the adsorption of Cu (II) using existing biological materials with high adsorption efficiency, abundant resources, environmental friendliness and low price have attracted great interest, such as various types of cellulose [12], crustaceans [13] and lignin [14].

In China, up to 10,000 tons of shrimp shells are produced every year. The shells are discarded as solid waste, which are both a waste of resources and a source of damage to the environment. Waste shrimp shells usually contain residue proteins, which can be regarded as the substrate for the incubation of bacteria. In addition, the landfill of the waste shrimp shells would contribute to the leachate containing a high amount of COD, which would be environmentally detrimental in the form of water and oil pollution. If waste shrimp shells can be effectively utilized in the production practices, their economic benefits will be greatly enhanced and environmental pollution will be minimized [15,16]. The resource utilization of waste shrimp shells is usually performed through chemical and physical modification into chitosan as the main component of biomolecular materials, with a large number of active sites such as amino (-NH_2_) and hydroxyl (-OH) groups, which are known to interact with metal cations such as Cu (II) through chelation and electrostatic attraction. Additionally, modified waste shrimp shells also have stable chemical properties, a large specific surface area and porous structures, and thus are considered ideal biomass adsorbents for Cu (II) [9,13,17,18]. Recently, an adsorbent was prepared by reaction of waste shrimp shells with hydrochloric acid reagent, achieving a maximum adsorption capacity of 0.38 mmol/g at pH = 4 [19]. In another study, waste shrimp shell, bovine cortical bone and snail shell were the raw materials used to prepare the powder particle adsorbent. At pH = 5.5, the maximum adsorption capacity was 0.39 mmol/g [20]. Moreover, 1-butyl-3-methylimidazolium acetate and γ-valerolactone were used as co-solvents in preparing thin films of waste shrimp shells mixed with sulfate lignin, achieving a maximum adsorption capacity of 0.63 mmol/g for Cu (II) [21]. Although the above methods are simple to treat and modify the waste shrimp shells, the adsorption capacity of Cu (II) is low. Moreover, Zhang et al. prepared magnetic MnFe_2_O_4_/CS microspheres with waste shrimp shells and MnFe_2_O_4_ as raw materials and pentanethylene glycol as the cross-linking agent for Cu (II) removal, with a maximum adsorption capacity of 0.98 mmol/g [17]. Le et al. extracted substances in green tea as cross-linking agents to prepare magnetic chitosan particles (MCPs) from waste iron residue and waste shrimp shells, and the results showed that the maximum adsorption capacity of Cu (II) reached 2 mmol/g [6]. It is worth noting that the adsorption capacity of Cu (II) can be improved by modifying waste shrimp shells in various ways. However, from the perspective of sustainable development and environmental protection, these modification steps are usually cumbersome and complex, since they are very expensive. Moreover, these modified reagents are more toxic and non-reusable, which easily brings about environmental problems such as resource waste and secondary pollution. Therefore, it is desirable to produce the adsorbent and run the adsorption process in a way that is beneficial to the environment, achieving a decent adsorption capacity to remove the pollutant.

Generally speaking, the Cu (II) adsorbed on the waste shrimp shell is almost impossible to degrade, and is easy to pollute during storage and transportation, causing severe environmental pollution [17,22]. The separation and recovery of Cu (II) from adsorbents to control heavy metal pollution are gradually becoming the focus of the current research [1,23,24]. The major reason for desorption is the removal of Cu (II) from waste shrimp shells, so that the waste shrimp shells can be reused while the Cu (II) can be recovered [25]. Currently, many researchers have focused on the adsorption of heavy metals by adsorbents, but few have focused on the systematic desorption of adsorbents. As a result of the poor thermal stability and biodegradability of the adsorbents prepared from waste shrimp shells, chemical methods are the best for desorption. According to the literature reports, acid reagents such as HCl, EDTA and HNO_3_ were used to desorb Cu (II), with desorption efficiencies reaching 94%, 93% and 89%, respectively [26,27,28]. A systematic study on the experimental conditions for the desorption of waste shrimp shells should be carried out. In fact, the effects of using different desorption reagents on the separation and recovery of Cu (II) from waste shrimp shells under different experimental conditions also vary greatly. Therefore, it is important to carry out systematic desorption experiments, which would determine the best desorption reagent and desorption reaction conditions, achieving the best desorption effects [1,29]. In addition, the reuse of the modification reagents is also important, but the existing literature hardly mentions the reuse of the modified reagents [8,9]. If the reuse of the modification reagent can be realized, it will cut the costs of adsorbent preparation and improve the utilization efficiency of resources.

In this research, for the first time, cheap and easily available NaOH solution was utilized as the modification reagent and waste shrimp shells were the raw materials selected in the preparation of waste shrimp shell adsorbents by a simple one-step direct modification, and the adsorption efficiency of Cu (II) in the solution was studied. To the best of our knowledge, there are few reports on the preparation of heavy metal adsorbents by the direct modification of waste shrimp shells with one type of NaOH solution. Specifically, the possibility of reusing NaOH waste alkali solution in the preparation of waste shrimp shell adsorbents was explored for the first time. With the basis of green chemical synthesis and efficient recycling of resources, this provides a new way to recycle resources. Moreover, to explore the renewable and stable use of waste shrimp shells in the process of adsorption–desorption, systematic desorption experiments were carried out to explore the effects of different desorption agent types, temperature, time and other factors on the desorption performance of waste shrimp shells, to screen for the best desorption reagent and experimental reaction conditions. 

## 2. Results and Discussion

### 2.1. Results and Analyses of Orthogonal Experiment

Orthogonal experimental design is an important mathematical method to study multi-factor experiments, and it is also a reasonable and effective arrangement for the experimental factors so as to achieve optimum results with the minimum number of experiments [30]. Table 1 displays the effects of concentration of NaOH, solid–liquid ratio and reaction time on the preparation of MS. In view of the orthogonal analyses of Table 1, the values of Σy/3 and Δ have been calculated by statistical software. The factors influencing the adsorption efficiency of Cu (II) were listed in a decreasing order as follows: B > C > A according to the Δ value. The maximum adsorption efficiency of Cu (II) was acquired when the concentration of NaOH, the solid–liquid ratio and the reaction time were B2C3A2 (solid–liquid ratio: 60:1, reaction time: 12 h, NaOH: 1%). Additionally, the solid–liquid ratio was the most important determinant of the adsorption efficiency of Cu (II) according to the Δ value. To save the cost of production and time under the concentration range, the optimum conditions were as follows: B2C2A2 (solid–liquid ratio: 60:1, soaking time: 8 h, NaOH: 1%), and the adsorption efficiency of Cu (II) could reach 22.17%.

### 2.2. Structure and Morphology Characterization

#### 2.2.1. FTIR Analysis

**Figure 1:**
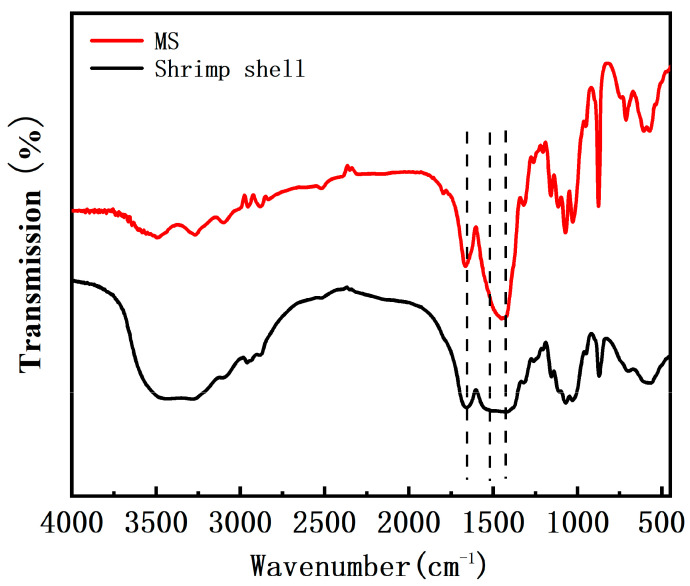
FTIR spectra of shrimp shell and MS.

Figure 1 displays the FTIR spectra of the shrimp shell and MS treated by 1% NaOH. Compared with the shrimp shell, the MS showed an associated O–H stretching vibration with a strong absorption peak at 3450 cm^−1^, an N–H stretching vibration absorption peak near 3130 cm^−1^ and at 2927 and 2895 cm^−1^ assigned to -CH stretching vibrations in -CH and -CH_2_, which indicated that many amine groups were exposed to the surface of the MS. It is found that the vibration peak at 1575 cm^−1^ corresponding to -NH-CO- groups was obvious in shrimp shell [31], while it was significantly reduced in MS, indicating the hydrolysis of the amide groups of the shrimp shells in the presence of the NaOH solution. In addition, the peaks at 1450 and 1665 cm^−1^ were greatly strengthened in MS, as compared to those in the shrimp shell, corresponding to -NH deformation and bending vibrations in -NH_2_, respectively [32], indicating the generation of more -NH_2_ groups through the hydrolysis of the -NH-CO- groups of the shrimp shell. Therefore, the FTIR results indicated the successful reaction of the shrimp shell with the NaOH solution to generate more amine (-NH_2_) groups in the modified shrimp shell [33].

#### 2.2.2. Elemental Analysis

Shrimp shells mainly contain chitin, calcium carbonate, protein and other substances [16]. According to the elemental analysis, the contents of N(%), C(%), H(%) and S(%) are displayed in Table 2. The results illustrate that all four elements in MS are reduced by 37.6%, 17.2%, 15.5% and 88.8%, respectively, compared with those of the shrimp shells. It is known that the S and N are mainly contained in the shrimp shell’s protein [3]. Therefore, it can be inferred that most of the proteins in the shrimp shell are removed after the modification of 1% NaOH.

#### 2.2.3. SEM Analysis

**Figure 2:**
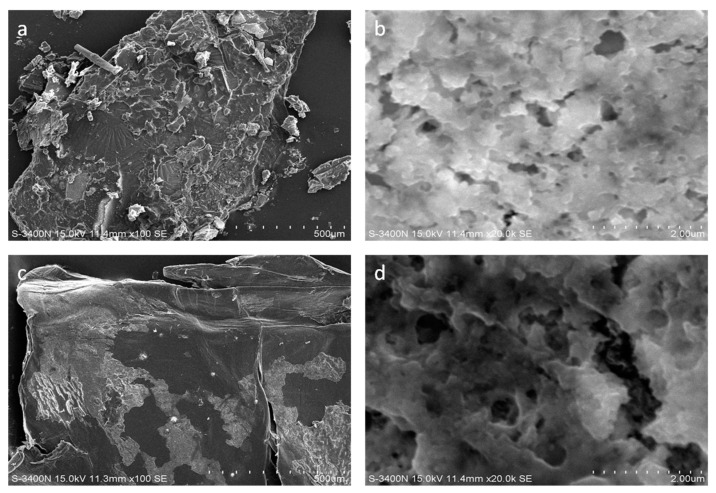
SEM morphology of the shrimp shell (**a**,**b**) and MS (**c**,**d**).

Figure 2 shows the SEM images of shrimp shell and MS. Figure 2a shows that the shape of the shrimp shell has an irregular geometry, with more small chips and uneven shapes, showing many small “hills” with uneven shapes, while MS is smooth and most of the protein and fat has been eliminated because of the treatment of the 1% NaOH from Figure 2c. Additionally, comparing Figure 2d with Figure 2b, the structure of the MS showed a distinct network structure and obvious porosity, suggesting that most of the protein and fat on the surface has been removed.

### 2.3. Effects of Experimental Conditions on Adsorption of Cu (II)

**Figure 3:**
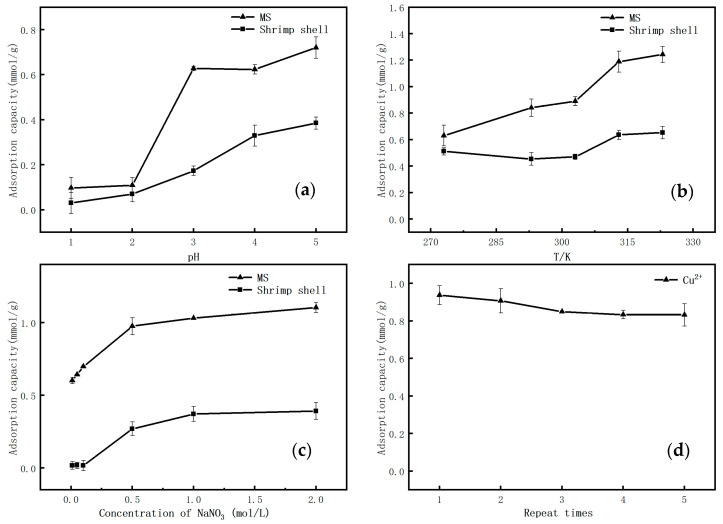
The effect of (**a**) pH (1–5); (**b**) temperature (273 K, 293 K, 303 K, 313 K and 323 K); (**c**) ionic strength (NO_3_^−^); and (**d**) 5-cycle times of 1% NaOH on the adsorption of Cu (II) by the MS.

To determine the optimum parameters for the adsorption process, the effects of different adsorption conditions of Cu (II) on the MS were investigated. The experimental results are presented in Figure 3. As shown in Figure 3a, the adsorption capacity of Cu (II) by MS increased with an increase in the pH from 1 to 5. At low pH (<2), the adsorption capacity of Cu (II) was low, while at pH (>3) the adsorption capacity increased from 0.11 mmol/g to 0.63 mmol/g, which is a significant increase of nearly six times. When the pH = 5, the adsorption capacity of MS reached its maximum at 0.72 mmol/g. At a lower pH, -NH_2_ would be protonated into the positively charged -NH_3_^+^, causing electrostatic repulsion between the Cu (II) ion and MS, which is not conducive to the adsorption of Cu (II). At pH = 5, the concentration of H^+^ in the solution is much lower, and hence the protonation of -NH_2_ is greatly reduced, thus significantly increasing the Cu (II) ion adsorption capacity [34,35].

The adsorption process is affected by heat change, and temperature is one of the important influencing factors. The effect of temperature on the adsorption capacity of Cu (II) was also investigated. The adsorption capacity increased with the increase of temperature, which improved from 0.63 to 1.23 mmol/g with the temperature increase from 293 K to 323 K, respectively, indicating the endothermic process of the adsorption reaction [36].

Figure 3c presents the influence of ionic strength on the adsorption capacity. With an increasing concentration of NO_3_^−^, the adsorption capacity of Cu (II) showed an increasing trend. It can be seen that the adsorption capacity of Cu (II) could reach 1.10 mmol/g when the concentration of NO_3_^−^ increased up to 2 mol/g, while the unmodified shrimp shell is very small, almost negligible. The main reason is that the reaction system contains partially protonated -NH_3_^+^. Under strong NO_3_^−^ concentrations, more charge balanced ions surrounded the adsorption sites with opposite charge (-NH_3_^+^), thereby neutralizing part of the positive charge at the adsorption sites, weakening the electrostatic repulsive force between the modified shrimp shells and Cu (II) and thus promoting the adsorption. Therefore, when existing electrostatic repulsion occurs between adsorbents and the adsorbed substance, the enhancement of ionic strength is beneficial to adsorption [37].

Repeatedly use of NaOH is an important way to reduce the cost of operation and save resources. The used 1% NaOH was filtered and separated from the modified shrimp shells, and the recovered waste NaOH was utilized again in modifying the waste shells, recycled for five times. Figure 3d displays the results representing the adsorption capacity of the Cu (II). It can been seen that the MS’s adsorption capacity of Cu (II) reached 0.93 mmol/g at the first time. The same procedure was applied for each cycle, and after the five successive runs, the adsorption capacity of Cu (II) was efficient and stable, maintaining tendency in 0.83–0.85 mmol/g. Therefore, it was determined that the modification of the shrimp shell with the NaOH solution that was reused several times (>5) was promising in metal treatments, which brings about a new consideration for the resource utilization of the exhausted NaOH solution from industries. The preparation of adsorbents from the reused waste NaOH solution reflects the “recycling” link of “production-consumption-recycling”, and enhances the recycling utilization rate of resources. For instance, waste NaOH solution generated in the dyeing factory can be used as a modifier to treat waste shrimp shells, achieving the purpose of turning waste into treasure and improving the utilization rate of waste NaOH solution and waste shrimp shells.

### 2.4. The Adsorption Kinetics of Cu (II) on MS

Based on the literature, the study of adsorption kinetics can provide important information about the adsorption mechanism of heavy metals on natural adsorbents. In this paper, pseudo-first-order and second-order were examined for the adsorption of Cu (II) on the MS. The results displayed in Table 3 indicated that the correlation coefficients (R^2^) based on pseudo-second-order kinetics are near unity for Cu (II), and the fitted equilibrium adsorption capacity of the Cu (II) corresponds with the experimental data as well. Therefore, the adsorption mechanism followed the pseudo-second-order model and the reaction was a process of chemical adsorption, with the migration of Cu (II) from the solution to the surface of the MS mainly through chelation.

### 2.5. The Adsorption Isotherms of Cu (II) on MS

**Figure 4:**
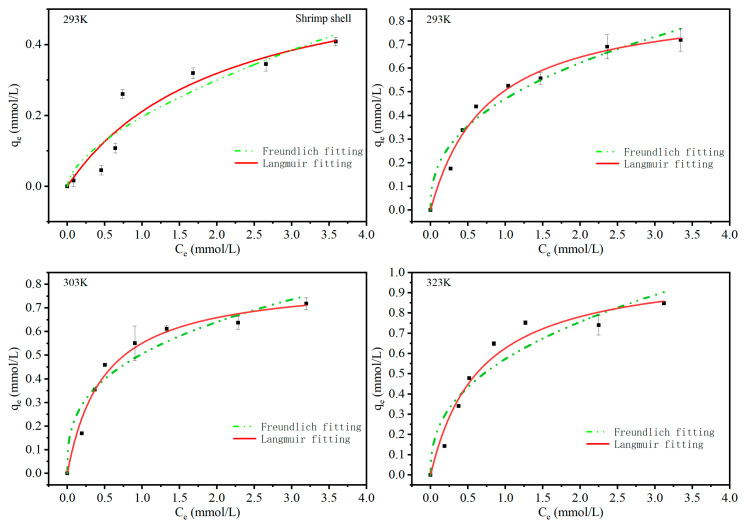
Langmuir and Freundlich fitting of adsorption isotherms for Cu (II) on shrimp shell at 293 K and MS at 293 K, 302 K and 323 K.

Langmuir and Freundlich isotherm models were utilized in the investigation of the features of the adsorption process. The constants and correlation coefficients of the isotherm equation are expressed in Table 4 and the isotherms plot is illustrated in Figure 4. Due to this, the correlation coefficients (R^2^) obtained from the Langmuir and Freundlich isotherm models for the adsorption of Cu (II) on MS were found to be 0.9706, 0.9928, 0.9425 and 0.9194, 0.8740, 0.8471 at 293 K, 302 K and 323 K, respectively, and this indicates that, under the concentration range studied, the Langmuir isotherm model gives much better fit than the Freundlich model, probably suggesting that the adsorption was mainly monolayer on a homogeneous adsorbent surface. The maximum adsorption capacity of Cu (II) on MS was 1.0403 mmol/g at 323 K, which can be attributed to the the monolayer adsorption via the coordination of -NH_2_ of the MS with Cu (II). The maximum adsorption capacity was better than that of waste shrimp shells treated with hydrochloric acid (0.38 mmol/g) [19] and the adsorbent prepared with shrimp shells, bovine cortical bones and snail shells (0.39 mmol/g) [20]. It was also superior to the MnFe_2_O_4_/CS microsphere adsorbent (0.98 mmol/g) [22].

### 2.6. Effects of Experimental Conditions on Desorption of Cu (II)

**Figure 5:**
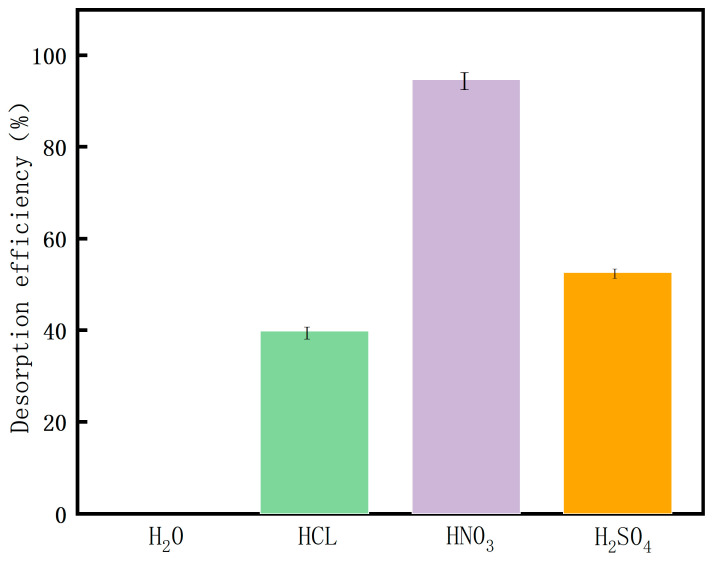
Influence of desorption solution types on Cu (II) desorption.

Figure 5 displays the effects of various desorption agents on the desorption of Cu (II). It can be seen from the figure that, under the same experimental conditions, the desorption rate of HNO_3_ is 94.69%, followed by H_2_SO_4_ with a desorption rate of 52.58% and HCl with a desorption rate of 39.85%. Therefore, HNO_3_ was a better desorption agent in this desorption experiment.

#### 2.6.1. The Effect of Concentration of Desorbent on the Desorption Efficiency of Cu (II)

**Figure 6:**
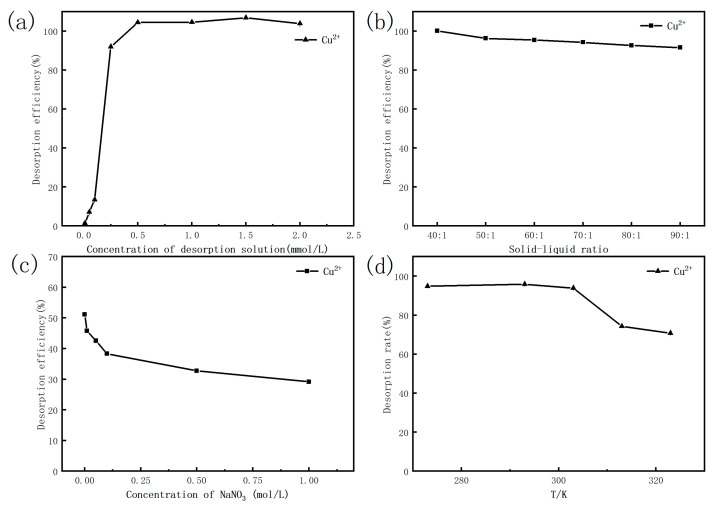
The effect of (**a**) concentration of desorbent (mol/L); (**b**) solid–liquid ratio; (**c**) ionic strength; and (**d**) temperature on the adsorption of Cu (II) by the MS.

The results of Figure 6a show that the Cu (II) desorption with HNO_3_ was extremely low with a concentration lower than 0.1 mol/L, while the Cu (II) desorption efficiency of 91.92% was obtained when 0.25 mol/L of the desorbent was used. Additionally, the Cu (II) desorption efficiency of 99 ± 1% could be reached with the range of 0.5–2 mol/L of the HNO_3_ solution, indicating that the Cu (II) was nearly removed from the MS. Taking into account the cost of the operation and desorption efficiency, it was advisable to achieve a better desorption efficiency at a lower concentration of the desorbent. Therefore, the most appropriate desorption concentration is 0.25 mol/L.

#### 2.6.2. The Effect of Solid-Liquid Ratio on the Desorption Efficiency of Cu (II)

The effect of different ratios of solid–liquid (MS / HNO_3_ solution) is presented at Figure 6b. The Cu (II) desorption of 100% was achieved at the ratio of 40:1. Upon the increase in the ratio, the desorption efficiency began to slowly decrease. Within the range of 50:1 and 60:1, the Cu (II) desorption was still above 95%. Therefore, to achieve high desorption efficiency under the same desorbent solution dosage, a solid–liquid ratio of 60:1 was confirmed.

#### 2.6.3. The Effect of Ionic Strength on the Desorption of Cu (II)

The effect of the ionic strength on the desorption of Cu (II) was investigated with the 0.25 mol/L of HNO_3_ as the desorbent and the 60:1 as the solid–liquid ratio, and the results are displayed in Figure 6c. It can be seen that as the NaNO_3_ concentration increased from 0 to 1 mol/L, the desorption efficiency of Cu (II) decreased from 51% to 28%, showing that the NaNO_3_ concentration had a definite inhibitory effect on the desorption of Cu (II) under the concentration range studied. The main reason is that the modified shrimp shell and Cu (II) formed a stable chelate in the adsorption process. In the desorption reaction, when NO_3_^−^ is added to the system, the reaction process goes in the opposite direction, thereby inhibiting the desorption of Cu (II).

R-NH_2_ --- Cu^2+^(NO_3_^−^)_2_ + H^+^ → R-NH_3_ + (NO_3_^−^) + Cu^2+^ + NO_3_^−^

where R-NH_2_ denotes those sites adsorbed with copper ions. Additionally, in a more acidic environment, a higher concentration of H^+^ will form more R-NH_3_^+^, which is not conducive to the adsorption of Cu (II). In the literature, many desorption studies were only performed under a specific condition. The results presented here show the importance of looking into the effect of the desorption conditions on the desorption efficiency, especially for metal ion desorption.

#### 2.6.4. The Effect of Temperature on the Desorption of Cu (II)

Figure 6d shows the effect of temperature on the desorption of Cu (II). The desorption efficiency of the MS for Cu (II) kept a stable range of 94–96% with temperature from 0–30 °C, while the efficiency rapidly decreased to under 70% when increasing the temperature from 303 K to 323 K. This accounts for the exothermic nature of the adsorption process, which is favorable at low temperatures.

#### 2.6.5. The Desorption Kinetics

**Figure 7:**
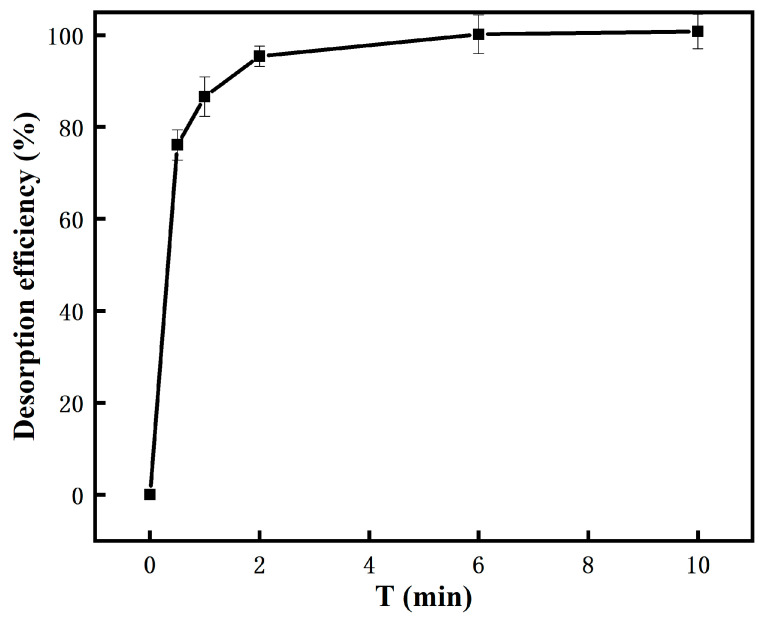
Desorption kinetics of Cu (II).

Figure 7 shows the desorption kinetics of Cu (II). The desorption rate of Cu (II) reached 76.09% in the first 30 s and 86.56% in 1 min. When the desorption time reached 6 min, the desorption rate reached 100%, indicating a very fast desorption kinetics.

### 2.7. Comparison of MS and Other Biomass Adsorbents

The preparation of heavy metal adsorbents from waste shrimp shells has become an area of focus by researchers. Modification of this kind of biomolecular material can increase the number of active sites in the structure to enhance the adsorption of heavy metals. Table 5 compares the studies on the enhancement of Cu (II) adsorption capacity through different modification reagents and modification processes when shrimp shells are used as raw materials. Compared with the modification methods in other studies, the modification steps in this study are simpler. In addition, the modification reagent in this study is economical, green, and easily obtained, and the re-use effect is significant. It is worth noting that the other five studies did not involve the exploration of the re-use of the modified reagent. In this work, the maximum adsorption capacity of the modified shrimp shell could reach 1.04 mmol/g and the systematic desorption experiments were carried out to explore the best desorption experimental conditions to improve the recycling efficiency of heavy metals.

## 3. Materials and Methods

### 3.1. Materials

Shrimp shell, *Metapenaeus ensis*, was readily available at the local market. Sodium hydroxide (NaOH, analytical reagent (AR)) was purchased from Tianjin Yongda Chemical Reagent Factory (Tianjin, China). Copper nitrate (Cu(NO_3_)_2_, AR) and ethylenediamine (EDA, AR) were bought at Shanghai Qiangshun Chemical Reagent Co., Ltd. (Shanghai, China). Sodium nitrate (NaNO_3_, AR) was purchased from Sinopharm Chemical Reagent Co., Ltd. (Beijing, China). Copper sulfate (CuSO_4_, AR) and copper chloride (CuCl_2_, AR) were purchased from Tianjin Tianli Chemical Reagent Co., Ltd. (Tianjin, China). The deionized water (18.25 MΩ*cm) is the product from an ultrapure water system (UPR-II-10T, Ulupure Technology Co., Ltd., Chengdu, China). All commercially available chemicals and reagents were used as received.

### 3.2. Modification and Preparation of the Shrimp Shell

A total of 150 g of the fresh shrimp shells was cleaned to remove the remaining attached stuff using 250 mL D.I. water, boiled in water for 3 min, rinsed with deionized water and dried by a vacuum oven (BZF-50, Shanghai Boxun) at 323 K for 24 h, then crushed using a mortar and ready for use as bio-sorbent. Orthogonal experiments were carried out to explore the effect of NaOH concentration, reaction time and solid–liquid ratio on the modification of the shrimp shell (MS), to determine the most efficient modification conditions. MS was prepared as follows: a fully ground shrimp shell was placed into a beaker, with the concentration of NaOH (0.5%, 1%, 2%), solid–liquid ratio (40:1, 60:1, 80:1) and reaction time (4 h, 8 h, 12 h), and then stirred with a magnetic stirrer. Then, the supernatants were poured away, and the deionized water was poured in the beaker, which was subsequently placed in a shaker (318 K, 130 r/min). The deionized water was changed every 40 min to remove the remaining protein and fat, and this operation was repeated three times. Finally, the products were filtered by diaphragm vacuum pump (GM-0.33A, Tianjin Jinteng) and dried by a vacuum oven at 333 K for 24 h to obtain the final product of MS [40].

### 3.3. Characterizations

The surface morphology of the MS structure was identified using a scanning electron microscope (S-3400N (II), Hitachi, Japan), operated at a typical accelerating voltage of 15 kV. The samples were sputter-coated with gold for 40 s at 15 mA prior to the observations. The elemental content of MS was determined by element analyzer (Vario EL Cube, Frankfurt, Germany). Samples of around 2 mg were wrapped in a tin vial and combusted in the element analyzer for the analyses. The analyses of the functional groups of MS were performed using a Fourier transformed infrared spectrometer (FTIR-8300PCS, Kyoto, Japan), using KBr pellets (about 1.5 mg of sample in 200 mg of KBr) in the wavelength range of 400–4000 cm^−1^, and each sample was scanned 128 times at a resolution of 4 cm^−1^. The concentration of Cu (II) was determined by UV–Vis spectrophotometer (UV3200, Mapada, Shanghai, China) using ethylenediamine (EDA) as a chelating reagent and acetic acid–sodium acetate as the buffer solution with a pH of 5.6, and the absorbance value was measured at a wavelength of 547 nm. The equation of the calibration curve is: y = 0.063x − 0.00002, R^2^ = 0.9999.

### 3.4. Influencing Factors on Cu (II) Adsorption

Using the Cu (NO_3_)_2_ to simulate wastewater containing Cu (II), the initial pH (pH = 1, 2, 3, 4, 5) value of the solution was adjusted by 1 mol/L HCl solution. The experiments were conducted using different initial concentrations (0.1, 0.5, 0.75, 1, 2, 3 and 4 mmol/L), temperature (273 K, 293 K, 303 K, 313 K and 323 K) and ionic strength using NaNO_3_ (0.01, 0.05, 0.1, 0.5, 1 and 2 mmol/L). Then, the predetermined MS was added at a solid to liquid ratio of 2:1 in a beaker with magnetic stirring for 8 h. After that, the supernatant solution was filtered through a filter membrane with an appropriate amount of EDA added, and the concentration of Cu (II) was determined by UV–Vis spectrophotometer. Each experiment was duplicated. The equilibrium adsorption amount and removal efficiency could be calculated following Equations (1) and (2), respectively, using the mass balance [41,42]

(1) $$q_{e}=\frac{(C_{0}-C_{e})V}{m}$$

(2) $$\eta (\%)=\frac{(C_{0}-C_{e})V}{C_{e}}\times 100$$

where *C*_0_ and *C_e_* are the initial and final concentrations (mmol/L), V is the volume of aqueous solution (L) and m is the mass of MS (mg). *q_e_* is equilibrium adsorption capacity (mmol/g) and η is adsorption efficiency (%).

### 3.5. Adsorption Kinetics Experiments

Kinetic studies were conducted in batch experiments at the initial pH of 6 and the initial concentration of Cu (II) (4 mmol/L), and then the MS was added in a beaker at a solid to liquid ratio of 2:1 and stirred at a constant speed using a magnetic stirrer. 3 mL of aqueous sample were taken out at the time intervals of 0, 1, 2, 4, 6, 8, 10, 15, 20, 30, 45, 60, 120, 180, 240, 300, 360 and 420 min, respectively. To specifically describe the kinetic behavior of Cu (II) adsorption by MS, a pseudo-first-order kinetic model (Equation (3)) and a pseudo-second-order kinetic model (Equation (4)) were fitted to the data of the adsorption kinetics. Adsorption kinetic data are often analyzed with kinetic models to reveal whether an adsorption process is dominated by a physical or chemical adsorption. The formulas are as follows:

(3) $$\ln(Q_{e}-Q_{t})=-k_{1}t+\ln Q_{e}$$

(4) $$\frac{t}{Q_{t}}=\frac{1}{k_{2}Q_{e}^{2}}+\frac{t}{Q_{e}}$$

where *Q_e_* is equilibrium adsorption capacity (mmol/g); *Q_t_* is the adsorption capacity at time *t* (mmol/g); and k_1_ ((min^−1^)) and k_2_ (g/(mmol·min)) are the pseudo-first-order parameters and pseudo-second-order parameters, respectively.

### 3.6. Adsorption Isotherm Experiments

The Langmuir and Freundlich adsorption isotherms were used to study the adsorption capacity of the adsorbent between adsorbent and adsorbate.

(5) $$q_{e}=\frac{Q_{max}bC_{e}}{1+bC_{e}}$$

(6) $$q_{e}=K_{f}C_{e}^{1/n}$$

where *Q_max_* is the maximum adsorption capacity of adsorbent (mmol/g); *q_e_* is the equilibrium adsorption capacity (mmol/g); *C_e_* is the equilibrium concentration (mmol/L); and *b* is the equilibrium constant of adsorption in Langmuir isotherm model. The larger the value of *b*, the stronger the adsorption capacity. *K_f_* and *n* are the adsorption constants in Freundlich isotherm model, and when *n* > 1, it is preferential adsorption.

### 3.7. Desorption Experiments

The pre-determined amounts of exhausted adsorbents were placed in contact with different desorbents: HCl (0.1 mol/L), HNO_3_ (0.1 mol/L) and H_2_SO_4_ (0.05 mol/L), for a desorption time of 4 h. The desorption experiments were carried out using different concentrations of HNO_3_ (0.01, 0.05, 0.1, 0.25, 0.5, 1, 1.5 and 2 mmol/L), solid–liquid ratios (40:1, 50:1, 60:1, 70:1, 80:1 and 90:1), temperatures (273 K, 293 K, 303 K, 313 K and 323 K) and ionic strengths using NaNO_3_ solution (0.01, 0.05, 0.1, 0.5 and 2 mol/L). The amount of desorbed Cu (II) was determined as in the above description. According to the changes in concentration of Cu (II) in the solution before and after desorption, equilibrium desorption quantity and desorption efficiency can be calculated following Equations (7) and (8):

(7) $$D=\frac{C_{e}V}{m}$$

(8) $$E_{D}=\frac{D}{Q}\times 100$$

where D is the desorption capacity (mmol/g), *C_e_* is the equilibrium concentration (mmol/L), V is the volume of regeneration solution (mL) and m is the amount of saturated adsorbent (mg). *E_D_* is the desorption efficiency (%), D is the desorption capacity (mmol/g) and Q is the adsorption capacity (mmol/g).

## 4. Conclusions

A promising biomass adsorbent for the removal of Cu (II) was successfully prepared via facile and simple one-step modification with waste shrimp shells as the raw material and NaOH as the modification agent. The SEM, FTIR and elemental analysis indicate that a distinct structure containing -NH_2_ groups was present on the surface. Through the orthogonal experiments, it was determined that the best preparation conditions of the MS were that: the ratio of waste shrimp shell to NaOH material was 60:1, the concentration of NaOH was 1% and the reaction time was 8 h. The Langmuir and the pseudo-second-order models displayed better fitting to experimental data, which meant that the adsorption for Cu (II) was mainly due to the chelation. The maximum adsorption capacity of Cu (II) on MS was 1.04 mmol/g at 323 K. Significantly, the MS modified by the consecutive use of the waste NaOH solution (>5 times) showed the stable adsorption capacity of the Cu (II) (0.83–0.85 mmol/g,), which showed a new way to recycle resources. Furthermore, to explore the renewable and stable use of waste shrimp shells in the process of adsorption–desorption, systematic desorption experiments screened out the best desorption reagent and experimental reaction conditions: the desorption rate can reach 100% in 6 min with HNO_3_ as the desorption agent.

In conclusion, the MS prepared by simple one-step modification is an effective and cost-effective alternative to commercial materials for the removal of the Cu (II) from solution. Recycling the modifier such as waste NaOH solution can greatly reduce the cost of preparing adsorbents and improve the utilization efficiency of resources, achieving the purpose of turning waste into treasure and enhancing economic performance.

## Figures and Tables

**Table 1:** Results and analyses of the orthogonal experiment.

	A	B	C	D	E
1	0.5	40:1	12	0.5492	19.67
2	1	60:1	12	0.6611	23.68
3	2	60:1	4	0.6063	21.72
4	0.5	60:1	8	0.6190	22.17
5	1	40:1	4	0.5412	19.39
6	1	80:1	8	0.4230	15.15
7	0.5	80:1	4	0.3301	11.82
8	2	80:1	12	0.3619	12.96
9	2	40:1	8	0.56904	20.38
Σy	53.66	59.44	52.93		
Σy	58.22	67.57	57.7		
Σy	55.06	39.93	56.31		
Σy/3	17.89	19.81	17.64		
Σy/3	19.41	22.52	19.23		
Σy/3	18.35	13.31	18.77		
Δ	1.52	9.21	1.59		

A: the concentration of NaOH (%), B: solid–liquid ratio, C: reaction time (h), D: equilibrium adsorption capacity (mmol/g), E: adsorption efficiency of Cu (II) (%).

**Table 2:** Elemental analysis of N(%), C(%), H(%) and S(%).

	N(%)	C(%)	H(%)	S(%)
A	7.81 (±0.26)	36.04 (±1.40)	5.73 (±0.20)	0.47 (±0.08)
B	4.87 (±0.98)	29.83 (±1.80)	4.84 (±0.17)	0.05 (±0.003)

A: shrimp shell, B: MS.

**Table 3:** Kinetic parameters for the adsorption of Cu (II) on shrimp shell (A) and MS (B).

Pseudo-First-Order Kinetic					Pseudo-Second-Order Kinetic		
	*C*_0_ (mmol/L)	*q_e_* (mmol/g)	k_1_ (min^−1^)	R_1_^2^	*q_e_* (mmol/g)	k_2_ (g/(mmol·min))	R_2_^2^
A	4	0.76	0.0218	0.882	0.8716	0.0521	0.9804
B	4	0.49	0.0122	0.6804	0.5712	0.0432	0.9649

A: shrimp shell, B: MS.

**Table 4:** Langmuir and Freundlich constants and correlation coefficients of Cu (II) on shrimp shell and MS.

	T (K)	Langmuir			Freundlich		
		*Q_max_* (mmol/g)	*b* (mmol/L)	R^2^	*n*	*K_f_*	R^2^
Shrimp shell	293	0.4091	2.1612	0.8605	1.6332	0.1960	0.8714
MS	293	0.7200	3.1300	0.9706	2.4772	0.4707	0.9194
	303	0.8197	2.0495	0.9628	2.9273	0.5057	0.8740
	323	1.0403	1.2535	0.9425	2.5001	0.5732	0.8471

**Table 5:** Comparison of adsorption capacity of Cu (II) by different adsorbents.

Adsorbents	Modified Reagents	*Q_max_* (mmol/g)	Desorption Experiment	Modified Reagent Reuse	Ref.
Shrimp shell	HCl	0.38	-	-	[19]
Shrimp shell/nails/bone	HCl/NaOH	0.39	-	-	[20]
Shrimp shell/MnFeO4	HCl/NaOH Pentyleneglycol	0.98	Yes	-	[17]
Shrimp shell nanoparticles	Salicylaldehyde/tripolyphosphate	1.33	-	-	[38]
Shrimp shell Schiff base resin	MVE-alt-MA	1.32	Yes	-	[39]
Shrimp shell	NaOH	1.04	Yes	Yes	In this study

## Data Availability

The data presented in this study are available in the article.

## Sample Availability

Samples of the materials are available from the authors.

## References

[1] Vakili M., Deng S., Cagnetta G., Wang W., Meng P., Liu D., Yu G. (2019). Regeneration of chitosan-based adsorbents used in heavy metal adsorption: A review. Sep. Purif. Technol..

[2] Li C., Shi W., Shang L. (2019). Latent feature representation for cohesive community detection based on convolutional auto-encoder. Big Data.

[3] Fu Z., Xi S. (2020). The effects of heavy metals on human metabolism. Toxicol. Mech. Methods.

[4] Zwolak A., Sarzyńska M., Szpyrka E., Stawarczyk K. (2019). Sources of soil pollution by heavy metals and their accumulation in vegetables: A Review. Water Air Soil Pollut..

[5] Zhang L., Zeng Y., Cheng Z. (2016). Removal of heavy metal ions using chitosan and modified chitosan: A review. J. Mol. Liq..

[6] Thuan L.V., Chau T.B., Ngan T.T.K., Vu T.X., Nguyen D.D., Nguyen M.-H., Thao D.T.T., To Hoai N., Sinh L.H. (2017). Preparation of cross-linked magnetic chitosan particles from steel slag and shrimp shells for removal of heavy metals. Environ. Technol..

[7] Choi J.-W., Song M.-H., Bediako J.K., Yun Y.-S. (2020). Sequential recovery of gold and copper from bioleached wastewater using ion exchange resins. Environ. Pollut..

[8] Duarte-Nass C., Rebolledo K., Valenzuela T., Kopp M., Jeison D., Rivas M., Azócar L., Torres-Aravena Á., Ciudad G. (2020). Application of microbe-induced carbonate precipitation for copper removal from copper-enriched waters: Challenges to future industrial application. J. Environ. Manag..

[9] Urbina L., Guaresti O., Requies J., Gabilondo N., Eceiza A., Corcuera M.A., Retegi A. (2018). Design of reusable novel membranes based on bacterial cellulose and chitosan for the filtration of copper in wastewaters. Carbohydr. Polym..

[10] Hosain A.N.A., El Nemr A., El Sikaily A., Mahmoud M.E., Amira M.F. (2020). Surface modifications of nanochitosan coated magnetic nanoparticles and their applications in Pb (II), Cu (II) and Cd (II) removal. J. Environ. Chem. Eng..

[11] Jiang C., Wang X., Wang G., Hao C., Li X., Li T. (2019). Adsorption performance of a polysaccharide composite hydrogel based on crosslinked glucan/chitosan for heavy metal ions. Compos. Part B Eng..

[12] O’Connell D.W., Birkinshaw C., O’Dwyer T.F. (2008). Heavy metal adsorbents prepared from the modification of cellulose: A review. Biores. Technol..

[13] Ali M.E.A., Aboelfadl M.M.S., Selim A.M., Khalil H.F., Elkady G.M. (2018). Chitosan nanoparticles extracted from shrimp shells, application for removal of Fe (II) and Mn (II) from aqueous phases. Sep. Sci. Technol..

[14] Szalaty T.J., Klapiszewski Ł., Jesionowski T. (2020). Recent developments in modification of lignin using ionic liquids for the fabrication of advanced materials—A review. J. Mol. Liq..

[15] Khademian E., Salehi E., Sanaeepur H., Galiano F., Figoli A. (2021). A systematic review on carbohydrate biopolymers for adsorptive remediation of copper ions from aqueous environments—Part B: Isotherms, thermokinetics and reusability. Sci. Total Environ..

[16] Mathew G.M., Mathew D.C., Sukumaran R.K., Sindhu R., Huang C.-C., Binod P., Sirohi R., Kim S.-H., Pandey A. (2020). Sustainable and eco-friendly strategies for shrimp shell valorization. Environ. Pollut..

[17] Zhang Y., Wang Y., Zhang Z., Cui W., Zhang X., Wang S. (2020). Removing copper and cadmium from water and sediment by magnetic microspheres-MnFe_2_O_4_/chitosan prepared by waste shrimp shells. J. Environ. Chem. Eng..

[18] Shekhawat A., Kahu S., Saravanan D., Jugade R. (2017). Removal of Cd (II) and Hg (II) from effluents by ionic solid impregnated chitosan. Int. J. Biol. Macromol..

[19] Maachou H., Bal Y., Chagnes A., Cote G. (2019). Copper sorption on chitin and acid-washed shrimp shells from *Palinurus elephas*: Isotherm and kinetic studies. Int. J. Environ. Sci. Technol..

[20] Bambaeero A., Bazarganlari R. (2020). Simultaneous removal of copper and zinc ions by low cost natural Snail shell/hydroxyapatite/chitosan composite-ScienceDirect. Chin. J. Chem. Eng..

[21] Duan Y., Freyburger A., Kunz W., Zollfrank C. (2018). Lignin/chitin films and their adsorption characteristics for heavy metal ions. ACS Sustain. Chem. Eng..

[22] Abali M., Ait Ichou A., Zaghloul A., Sinan F., Zerbet M. (2020). Removal of nitrate ions by adsorption onto micro-particles of shrimp-shells waste: Application to wastewater of infiltration-percolation process of the city of Agadir (Morocco). Mater. Today Proc..

[23] Khademian E., Salehi E., Sanaeepur H., Galiano F., Figoli A. (2020). A systematic review on carbohydrate biopolymers for adsorptive remediation of copper ions from aqueous environments-part A: Classification and modification strategies. Sci. Total Environ..

[24] Momina M., Mohammad S., Suzylawati I. (2018). Regeneration performance of clay-based adsorbents for the removal of industrial dyes: A review. RSC Adv..

[25] Zanella O., Tessaro I.C., Féris L.A. (2015). Desorption- and decomposition-based techniques for the regeneration of activated carbon. Chem. Eng. Technol..

[26] Osifo P.O., Neomagus H.W.J.P., Everson R.C., Webster A., vd Gun M.A. (2009). The adsorption of copper in a packed-bed of chitosan beads: Modeling, multiple adsorption and regeneration. J. Hazard. Mater..

[27] Ngah W.S.W., Fatinathan S. (2010). Adsorption characterization of Pb (II) and Cu (II) ions onto chitosan-tripolyphosphate beads: Kinetic, equilibrium and thermodynamic studies. J. Environ. Manag..

[28] Laus R., de Fávere V.T. (2011). Competitive adsorption of Cu (II) and Cd (II) ions by chitosan crosslinked with epichlorohydrin–triphosphate. Biores. Technol..

[29] Igberase E., Ofomaja A., Osifo P.O. (2019). Enhanced heavy metal ions adsorption by 4-aminobenzoic acid grafted on chitosan/epichlorohydrin composite: Kinetics, isotherms, thermodynamics and desorption studies. Int. J. Biol. Macromol..

[30] Wang J.-P., Wang J.P., Chen Y.Z., Yuan S.J., Sheng G.P., Yu H.Q. (2009). Synthesis and characterization of a novel cationic chitosan-based flocculant with a high water-solubility for pulp mill wastewater treatment. Water Res..

[31] Mohanasrinivasan V., Mishra M., Paliwal J.S., Selvarajan E., Devi C.S. (2014). Studies on heavy metal removal efficiency and antibacterial activity of chitosan prepared from shrimp shell waste. 3 Biotech.

[32] Li N., Bai R. (2005). A novel amine-shielded surface cross-linking of chitosan hydrogel beads for enhanced metal adsorption performance. Ind. Eng. Chem. Res..

[33] Nan L., Ba I.R. (2006). Highly enhanced adsorption of lead ions on chitosan granules functionalized with Poly (acrylic acid). Ind. Eng. Chem. Res..

[34] Saad A.H.A., Azzam A.M., El-Wakeel S.T., Mostafa B.B., Abd El-latif M.B. (2018). Removal of toxic metal ions from wastewater using ZnO@Chitosan core-shell nanocomposite. Environ. Nanotechnol. Monit. Manag..

[35] Jin X., Li K., Ning P., Bao S., Tang L. (2017). Removal of Cu (II) ions from aqueous solution by magnetic chitosan-tripolyphosphate modified silica-coated adsorbent: Characterization and mechanisms. Water Air Soil Pollut..

[36] Kannamba B., Reddy K.L., AppaRao B.V. (2010). Removal of Cu (II) from aqueous solutions using chemically modified chitosan. J. Hazard. Mater..

[37] Dinu M.V., Dragan E.S. (2010). Evaluation of Cu^2+^, Co^2+^ and Ni^2+^ ions removal from aqueous solution using a novel chitosan/clinoptilolite composite: Kinetics and isotherms. Chem. Eng. J..

[38] Filius J.D., Lumsdon D.G., Meeussen J.C.L., Hiemstra T., Riemsdijk W.H.V. (2000). Adsorption of fulvic acid on goethite. Geochim. Cosmochim. Acta.

[39] Cegłowski M., Schroeder G. (2015). Preparation of porous resin with Schiff base chelating groups for removal of heavy metal ions from aqueous solutions. Chem. Eng. J..

[40] Kamari A., Ngah W.S.W. (2009). Isotherm, kinetic and thermodynamic studies of lead and copper uptake by H_2_SO_4_ modified chitosan. Colloid Surf. B Biointerfaces.

[41] Zhu Y., Fan W., Zhou T., Li X. (2019). Removal of chelated heavy metals from aqueous solution: A review of current methods and mechanisms. Sci. Total Environ..

[42] Huang Y., Wu H., Shao T., Zhao X., Peng H., Gong Y., Wan H. (2018). Enhanced copper adsorption by DTPA-chitosan/alginate composite beads: Mechanism and application in simulated electroplating wastewater. Chem. Eng. J..

